# Increasing incidence of hypotension in the emergency department; a 12 year population-based cohort study

**DOI:** 10.1186/s13049-016-0209-4

**Published:** 2016-03-02

**Authors:** Jon G. Holler, Daniel P. Henriksen, Søren Mikkelsen, Court Pedersen, Annmarie T. Lassen

**Affiliations:** Department of Emergency Medicine, Odense University Hospital, Odense, Denmark; Department of Respiratory Medicine, Odense University Hospital, Odense, Denmark; Department of Anaesthesiology and Intensive Care Medicine, Odense University Hospital, Odense, Denmark; Department of Infectious Diseases, Odense University Hospital, Odense, Denmark

**Keywords:** Hypotension, Epidemiology, Incidence, Etiology, Mortality, Emergency department

## Abstract

**Background:**

The epidemiology of hypotension as presenting symptom among patients in the Emergency Department (ED) is not clarified. The aim of this study was to describe the incidence, etiology, and overall mortality of hypotensive patients in the ED.

**Methods:**

Population-based cohort study at an University Hospital ED in Denmark from January 1, 2000, to December 31, 2011. Patients aged ≥18 years living in the hospital catchment area with a first time presentation to the ED with hypotension (systolic blood pressure (SBP) ≤100 mm Hg) were included.

Outcomes were annual incidence rates (IRs) per 100,000 person years at risk (pyar) and etiological characteristics by means of the International Classification of Diseases, Tenth Revision (ICD-10), as well as 7-day, 30-day, and 90-day all-cause mortality.

**Results:**

We identified 3,268 of 438,198 (1 %) cases with a mean overall IR of 125/100,000 pyar (95 % CI: 121–130). The IR increased 28 % during the period (from 113 to 152 cases per 100,000 pyar). Patients ≥65 years had the highest IR compared to age <65 years (rate ratio for men 6.3 (95 % CI: 5.6-7.1) and for women 4.2 (95 % CI: 3.6-4.9)). The etiology was highly diversified with trauma (17 %) and cardiovascular diseases (15 %) as the most common. The overall 7-day, 30-day and 90-day mortality rates were 15 % (95 % CI: 14–16), 22 % (95 % CI: 21–24) and 28 % (95 % CI: 27–30) respectively.

**Conclusion:**

During 2000–2011 the overall incidence of ED hypotension increased and remained highest among the elderly with a diversified etiology and a 90-day all-cause mortality of 28 %.

## Background

Systolic blood pressure (SBP) is widely used in the initial triage of acutely ill patients and forms a basic part of the initial assessment of the circulation [1]. The presence of hypotension often signifies critical illness and several large multicenter studies have used the presence of hypotension as an inclusion criteria together with other variables [2]. These studies often focus on highly selected hypotensive patient populations in specialized treatment units, and the evidence gained is a reflection of this selection.

During the past decade, research investigating annual trends in incidence rates (IR) of potentially hypotensive patients suggest opposite trends depending on the etiology and population of interest. While the annual IRs of sepsis seems to increase [3], the trend for myocardial infarction (MI) have decreased [4]. Whether population-based IRs and annual trends of primary undifferentiated hypotensive ED patients demonstrate dynamic trends, are not known. Previous estimates of hypotension in EDs rely mainly on hospital data samples that are weighted to extrapolate to national level estimates and are therefore vulnerable to sampling bias [5]. In general studies on this topic are limited, either by place of settings or selective inclusion criteria and conditions studied [6]. Population-based IRs of hypotension among ED patients is important to quantify as the presence of hypotension - even transient - is associated with worse outcomes and can therefore not be neglected [7]. The epidemiological knowledge gained, can serve as a foundation for future interventional studies in this critical population.

While ED visits in Denmark have been stable through recent years, ED visits among the ageing population are increasing [8]. Furthermore, time-sensitive critical illnesses (i.e. cardiogenic shock, severe sepsis, and the ‘golden hour’ of trauma), have increased the demand of prompt critical care recognition and delivery in the ED setting. Collectively, this adds to the hypothesis of a possible increasing trend in ED hypotension. We, therefore, conducted an ED populations-based cohort study to examine annual IRs in first time presentation of hypotension over a 12-year period from 2000–2011 and subsequent the etiology and short-term mortality.

## Methods

### Study design and setting

We conducted a population-based cohort study with data from the ED of Odense University Hospital, Denmark, during the period of 1th January 2000 to 31th December 2011 (12 years). Odense University Hospital is a 1,000-bed university teaching hospital representing all specialties including surgical, neurological, and general internal medical patients. The population served by this ED consists of four well-defined municipalities with a mixed rural–urban population of 290,000 persons. It is the only serving ED in this part of Denmark and provides primary 24-h acute medical care, with 48.000 annual visits.

### Selection of participants

Adults (age ≥ 18 years) were considered eligible when presenting to the ED with a SBP ≤ 100 mm Hg registered within 3 h upon arrival. Based on a recent published study, examining SPB thresholds and mortality in our ED, we defined a SBP ≤ 100 mm Hg as hypotension [9]. We used the Shock Index (SI) as a proxy for acute illness. SI is calculated as the ratio of heart rate to SBP and included as a categorical variable (<0.7, 0.7-1, ≥1) [10]. If a patient had multiple encounters with hypotension over the study period, only the first was included within the cohort. The primary date of contact defined the index date. Patients <18 years, patients residing outside the hospitals catchment area at the time of contact, and patients without a Danish personal identification number were excluded. We also excluded patients with a previous presentation of SBP ≤ 100 mm Hg. To minimize left sided censoring, patients who had visited the ED between 1 of January 1998 and 1 of January 2000 with hypotension were excluded as well. The background population, from which the cases were retrieved, was the composed general adult (≥18 years) Danish citizens living in the hospitals catchment area.

### Variables and outcome measures

The primary outcome was the IRs of hypotension (SBP ≤ 100 mm Hg) from 1st January 2000 to 31th January 2011 in the ED, both overall and by year. Secondary outcomes were etiological characteristics by means of major ICD-10 codes and the proportion of 7-day, 30-day, and 90-day all-cause mortality. The primary exposure variable was the first recorded SBP value at presentation.

SBP was measured with an automated oscillometric device or manual cuff and sphygmomanometer. HR was measured with ECG, palpation or pulse oximetry. We also included information on the additional covariates; age, gender and time of contact during the day (07:00–14:59, 15:00–22:59, 23:00–06:59). Charlson comorbidity index (CCI; 0, 1–2, >2) was used as a marker of comorbid illness.

We defined etiology based on the primary ED diagnoses and the immediate ensuing hospital discharge diagnosis. These were assigned by physicians in the ED at discharge/referral to other departments and based on the International Classification of Diseases, Tenth Revision (ICD-10) (see below).

### Data sources and processing

In Denmark every Danish citizen is assigned a unique 10-digit civil personal registry number (PRN-number). This unique PRN-number enables accurate linkage between the Danish national registers [11]. True population-based studies are hereby possible as all patient contacts are registered and linked between all Danish registries using the patients unique PRN-number.

#### The Danish national patient registry

Since 1995 the Danish National Patient Registry has been covering all in and out patient clinic contacts at hospitals in Denmark assembling data regarding dates of admission, discharge, admitting departments and all primary and secondary discharge diagnoses (ICD-10 code system) from hospitals (except psychiatric departments and hospitals) [11]. By discharge every unique patient journey is assigned one primary diagnosis and one or more secondary diagnosis (up to 20 diagnoses) classified according to the ICD-10 system. We used discharge diagnoses from the previous 10 years in order to generate a CCI for each enrolled patient upon the index contact date as a proxy for comorbid illness.

#### Database

Since 1996 all patients records from the ED are registered electronically and available as patients record notes from the contact. As a part of the routine procedure, all patients presenting to the ED, except those with minor orthopedic complaints, had their vital signs measured and registered by a nurse at arrival. The record notes are available in text-format, in which vital parameters are consistently stated, including time of admission and time of measured SBP and HR. By electronic screening it was possible to identify and retrieve information on all patients with a measured and registered SBP ≤100 mm Hg as well as the unique value of SPB and HR. The present data extraction process has been manually validated in 500 files with a sensitivity of 96 % (95 % CI [91–99]) and a specificity of 100 % (95 % CI [99–100]) for exact SBP, in the study by Kristensen et al. [9].

Data on municipality of residence, migration-, marital- and vital status, and date of birth were retrieved from The Danish Civil Registration System and linked to other registries and databases using the unique PNR-number [11].

#### Other registers and databases

We retrieved information regarding the annual mid-year population of persons 18 years old or older living in the hospitals catchment area (accessed September 2014 at Statistics Denmark website; http://www.statistikbanken.dk).

### Analysis

Baseline characteristics were presented as medians and interquartile range (IQR) for continuous variables and categorical variables as numbers and percentages. We used the Chi-square test for categorical variables and the Kruskal-Wallis equality-of-populations rank test for continuous variables. Patients were followed from index date until the date of death, completion of 90 days follow-up, emigration, or December 31, 2011, whichever came first.

The crude annual IRs were calculated as the number of IRs per 100,000 pyar (age ≥18 years) with the corresponding 95 % confidence intervals (95 % CI) assuming a Poisson distribution. The annual IRs were adjusted using direct standardization to the sex- and age distribution of the municipalities of the EDs catchment area midyear population in the year 2000. The population was defined as contributing to one person-year at risk per resident per year in the analyses. The incidence rates were estimated and analyzed using a Poisson regression model. Age group, gender, calendar time in years, and interaction between age group and gender were used in the adjusted model. Calender time was entered in the model as a continuous variable. Age was divided into two predefined age intervals: 18–64 years and ≥65 years. The Poisson model was assessed using the Hosmere Lemeshow goodness-of-fit test.

Etiological characteristics were categorized into major ICD-10 groups and calculated as frequencies and proportions based on primary registered conditions at discharge among all hypotensive patients as well as stratified into SBP intervals.

We constructed Kaplan-Meier curves and reported the all-cause 90-day mortality, stratified by SBP intervals. Comparisons between groups were evaluated with a log-rank test. Cuzick’s test was used for trends in mortality between SBP intervals. All tests of significance were two-tailed, and *p* values <.05 were considered significant. Missing values (ICD-codes; *n* = 2 and HR; *n* = 128) were excluded in the analysis of the specific variable. Statistical analyses were performed using Stata version 13.1 (Stata Corporation LP ®, Texas, USA).

### Ethics committee approval

The study was approved by the Danish Data Protection Agency (J.nr 2008-58-0035) and the Danish Health and Medicines Authority (j.nr. 3-3013-205/1). In accordance with Danish law, observational studies performed in Denmark do not need approval from the Medical Ethics Committee. The study was conducted according to the STROBE statement.

## Results

### Participants

**Fig. 1:**
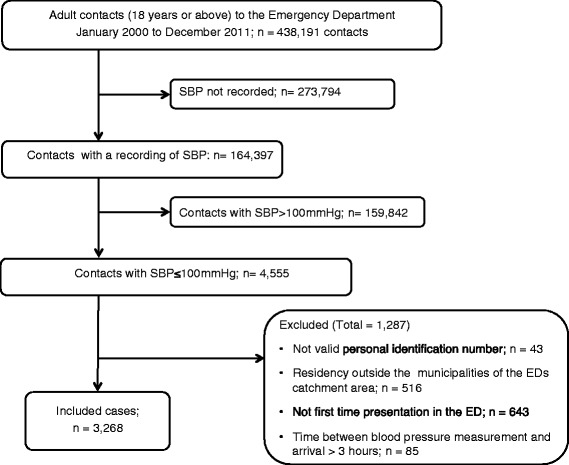
Flow chart of patients recruited to the study

**Fig. 2:**
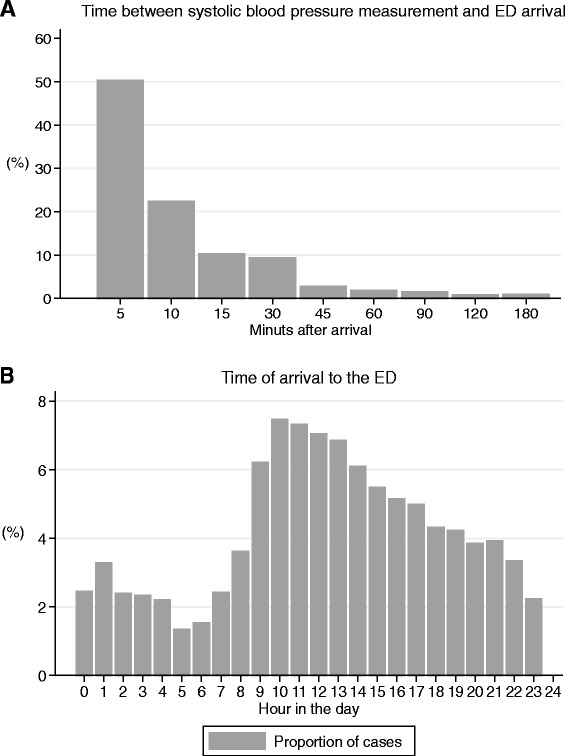
Histogram illustrating time between blood pressure measurement and arrival to the ED (**a**) and time of arrival stratified into hours of the day (**b**)

Among all patients the proportion of hypotensive patients was 1 % (4,555/438,198) (Fig. 1). After exclusions (see Fig. 1 for details) we included 3,268 patients with a presentation of hypotension for further analysis. Of these the median age of 68 (IQR, 50–80), 1,602 (49 %) were males and 858 (26 %) presented with a Charlson Comorbidity Index greater than 2 (Table 1). Median SBP was 91 (IQR, 84–96). A SBP between 90 and 100 mm Hg was present in 1,725 (53 %), 920 (28 %) had a SBP between 80 and 90 mm Hg and 623 (19 %) had a SBP below 80 mm Hg (Table 1). Most patients had their SBP measured within 30 min (92 %) after arrival and 50 % were measured within 5 min. Contacts showed a diurnal rhythm with most patients arriving during the day and evening (see Fig. 2a and b).

**Table 1:** Baseline characteristics at time of arrival to the ED^a^

Variable	Total (%) SBP ≤ 100 mm Hg	90 > SBP ≤ 100 mm Hg	80 > SBP ≤ 90 mm Hg	SBP ≤ 80 mm Hg
N (%)	3,268 (100 %)	1,725 (100 %)	920 (100 %)	623 (100 %)
Age in years, Median (IQR)	68 (50–80)	65 (45–79)	69 (54–80)	72 (58–82)
Sex (%)				
Male	1,602 (49 %)	792 (46 %)	462 (50 %)	348 (56 %)
Female	1,666 (51 %)	933 (54 %)	458 (50 %)	275 (44 %)
Age in age groups, yr (%)				
18–39	509 (16 %)	348 (20 %)	117 (13 %)	44 (7 %)
40–64	942 (29 %)	500 (29 %)	275 (30 %)	167 (27 %)
65–84	1,313 (40 %)	630 (37 %)	388 (42 %)	295 (47 %)
85+	504 (15 %)	247 (14 %)	140 (15 %)	117 (19 %)
Charlson Comorbidity Index (%)				
0	1,291 (40 %)	739 (43 %)	336 (37 %)	216 (35 %)
1 to 2	1,119 (34 %)	572 (33 %)	334 (36 %)	213 (34 %)
>2	858 (26 %)	414 (24 %)	250 (27 %)	194 (31 %)
Vital variables				
Systolic blood pressure, Median (IQR)	91 (84–96)	96 (93–97)	87 (84–89)	74 (66–79)
Diastolic blood pressure, Median (IQR)	55 (47–63)	60 (53–67)	54 (47–60)	44 (37–50)
Heart rate, Median (IQR)^b^	82 (68–100)	81 (68–96)	85 (68–104)	86 (68–104)
Shock Index (SI), n (%)				
SI, Median (IQR)	0.9 (0.8-1.2)	0.9 (0.7-1.0)	1.0 (0.8-1.2)	1.2 (0.9-1.5)
Time of contact				
Day (07:00–14:59)	1,536 (47 %)	779 (45 %)	462 (50 %)	295 (47 %)
Evening (15:00–22:59)	1,152 (35 %)	640 (37 %)	297 (32 %)	215 (35 %)
Night (23:00–06:59)	580 (18 %)	306 (18 %)	161 (18 %)	113 (18 %)
Mortality (Overall)				
7-day mortality, n (%)	489 (15 %)	150 (9 %)	153 (17 %)	189 (30 %)
30-day mortality, n (%)	731 (22 %)	264 (15 %)	233 (25 %)	234 (38 %)
90-day mortality, n (%)	922 (28 %)	363 (21 %)	286 (31 %)	273 (44 %)

^a^Values expressed as total number (fraction) and medians [25 percentile-75 percentile] as appropriat. Chi-squared test for categorical variables and Kruskal-Wallis test for continuous variables. ^b^128 observations missing

### Incidence rate

**Fig. 3:**
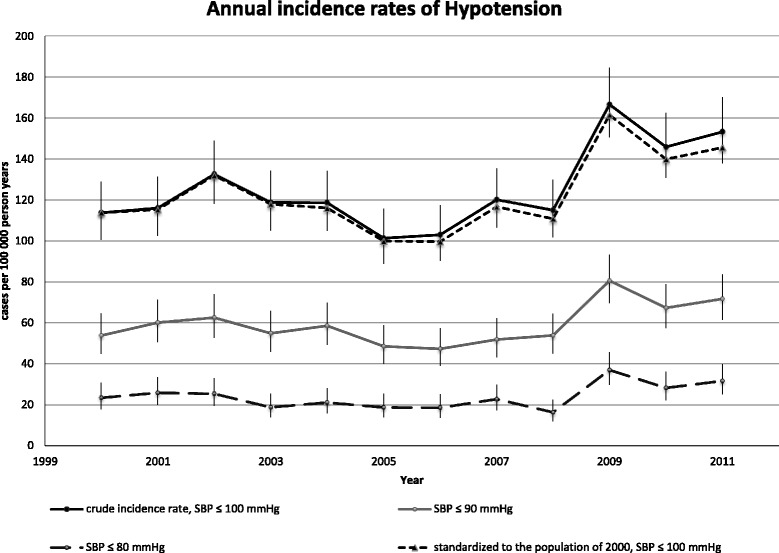
Annual incidence rate during 2000–2011. The crude annual incidence rates of hypotension from 2000 to 2011 and the standardized incidence rate to the population of the EDs cathment area in 2000 (using direct standardization on sex and ten-year age bands). Bars indicate the 95 % confidence interval based on a Poisson distribution. *P*-values represents Cuzick’s test for trend

**Fig. 4:**
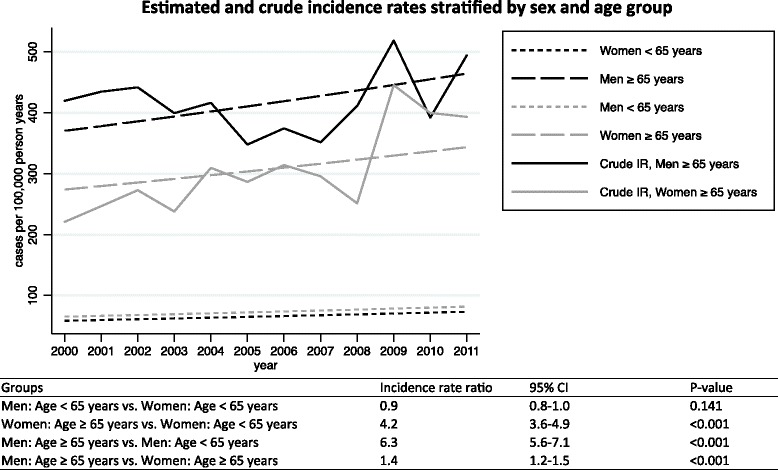
Estimated incidence rates and crude rates stratified by sex and age group from 2000 to 2011. Incidence rates estimated on the basis of a Poisson model adjusting for sex, age group, interaction between sex and age group, and calendar years. The table is showing the corresponding estimated incidence rate ratios with 95 % confidence intervals (95 % CI)

**Fig. 5:**
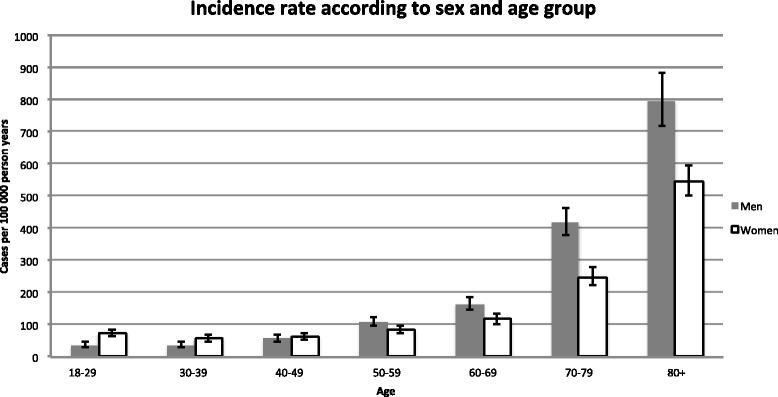
Crude incidence rates stratified by sex and age. Bars indicate the 95 % confidence interval based on a Poisson distribution

The annual crude IRs together with the standardized IRs of hypotension (SBP ≤100 mm Hg) and IRs of different levels of hypotension during the period 2000–2011 are shown in Fig. 3. The mean overall IR of hypotension during 2000–2011 was 125/100,000 pyar (95 % CI: 121–130). The corresponding standardized mean IRs estimates was 122/100,000 person-years at risk (95 % CI: 118–127). The overall IR increased by 28 % (95 % CI: 14-46 %) from 113 in 2000 to 152 in 2011, with an average adjusted annual increase of 2 % per year, (95 % CI: 1–3). Concordantly, the average annual increase using standardized estimates was 2 % (95 % CI: 1–3). The estimated IRs stratified by sex and age group with incidence rate ratios and crude IRs are shown in Fig. 4. Men aged ≥65 had a six-time higher IR than men aged <65 years. We observed increasing IRs during the years 2008–2011, preceded by more or less stable rates during 2000–2008. When stratified on SBP intervals the overall mean IR of 90> SBP ≤100 mm Hg was 66/100.000 pyar (95 % CI: 63–69) and 35/100,000 pyar (95 % CI: 33–37) in the interval 80 >SBP ≤ 90 mm Hg. SBP ≤80 mm Hg was 24/100,000 pyar (95 % CI: 22–26). Men aged 80+ had the highest incidence rate with 797/100,000 person-years at risk (95 % CI: 718–884). See Fig. 5.

### Etiology

The frequency and proportion of index and discharge conditions according to major *ICD-10* discharge diagnoses are presented in Table 2. The major overall discharge condition were coded as *“Injury, poisoning, and certain other consequences of external causes” (S00–T98)* (23 %) which were stratified into medical conditions *(T36-T69, T80-T98)* (8 %) and trauma *(S00-T35, T70-T79, T90-T99)* (17 %).*” Diseases of the circulatory system” (I00-I99)* (15 %), and *”Symptoms, signs, and abnormal clinical and laboratory findings, not elsewhere classified” (R00–R99)* (14 %). Analysis of discharge conditions across the period of observation revealed an increasing trend for infectious diseases (ICD10: A00–B99, *p*=0.003), respiratory diseases (ICD10: J00–J99: *p*=0.007), genitourinary system (ICD10: N00-N99, *p*=0.014), as well as symptoms, signs, and abnormal clinical and laboratory findings, not elsewhere classified (ICD10: R00-R99, *p* = 0.013) see Table 3.

**Table 2:** Frequency and proportion of primary admission conditions and discarge conditions according to major ICD-10 groups including SBP intervals^a^

		Total(%) SBP ≤ 100 mm Hg				90 > SBP ≤ 100 mm Hg		80 > SBP ≤ 90 mm Hg		SBP ≤ 80 mm Hg	
		Admission		Discarge		Discarge		Discarge		Discarge	
ICD-10 groups	Disease categories	n	%	n	%	n	%	n	%	n	%
S00–T98	Injury, poisoning, and certain other consequences of external causes^b^	805	25	756	23	458	27	192	21	106	17
	Medical (T36-T69, T80-T98)	186	6	201	6	115	7	59	6	27	4
	Trauma (S00-T35, T70-T79, T90-T99)	619	19	555	17	343	20	133	14	79	13
I00–I99	Diseases of the circulatory system	374	11	489	15	194	11	152	17	143	23
R00–R99	Symptoms, signs, and abnormal clinical and laboratory findings, not elsewhere classified	545	17	457	14	251	15	122	13	84	13
J00–J99	Diseases of the respiratory system	229	7	304	9	153	9	95	10	56	9
K00–K93	Diseases of the digestive system	209	6	295	9	130	8	98	11	67	11
Z00–Z99	Factors influencing health status and contact with health services	641	20	245	8	137	8	72	8	36	6
A00–B99	Certain infectious and parasitic diseases	63	2	150	5	69	4	38	4	43	7
E00–E90	Endocrine, nutritional, and metabolic diseases	156	5	135	4	75	4	39	4	21	3
F00–F99	Mental and behavioral disorders	107	3	129	4	90	5	25	3	14	2
N00–N99	Diseases of the genitourinary system	33	1	83	3	46	3	24	3	13	2
G00–G99	Diseases of the nervous system	42	1	63	2	41	2	16	2	6	1
D50–D89	Diseases of the blood and blood-forming organs and certain disorders involving the immune mechanism	21	1	53	2	28	2	14	2	11	2
C00–D48	Neoplasms	2	0	43	1	16	1	14	2	13	2
M00–M99	Diseases of the musculoskeletal system and connective tissue	28	1	37	1	23	1	8	1	6	1
L00–L99	Diseases of the skin and subcutaneous tissue	9	0	19	1	8	0	8	1	3	0
O00–O99	Pregnancy, childbirth, and the puerperium	2	0	7	0	3	0	3	0	1	0
Q00–Q99	Congenital malformations, deformations, and chromosomal abnormalities	0	0	1	0	1	0	0	0	0	0
Total		3,266	100	3,266	100	1,723	100	920	100	623	100

^a^2 observations missing, ^b^S00–T98 ICD-10 codes divided into trauma and medical conditions

**Table 3:** Frequency and approximate rounded proportions of discarge conditions according to major ICD-10 groups stratified by year

		Year													
		2000	2001	2002	2003	2004	2005	2006	2007	2008	2009	2010	2011	Total	P-trend^a^
ICD-10 groups		%	%	%	%	%	%	%	%	%	%	%	%	% (n)	
A00–B99	Certain infectious and parasitic diseases	1	5	8	5	6	5	9	11	8	14	12	16	100 (148)	0.003
C00–D48	Neoplasms	2	9	2	7	5	12	12	12	5	19	7	9	100 (43)	0.105
D50–D89	Diseases of the blood and blood-forming organs and certain disorders involving the immune mechanism	8	8	6	2	9	13	2	9	9	17	8	9	100 (53)	0.253
E00–E90	Endocrine, nutritional, and metabolic diseases	13	4	6	7	8	4	8	5	5	13	13	13	100 (135)	0.199
F00–F99	Mental and behavioral disorders	12	6	12	9	10	2	4	7	5	11	13	10	100 (129)	0.934
G00–G99	Diseases of the nervous system	5	5	10	10	11	6	11	5	10	13	8	8	100 (63)	0.301
I00–I99	Diseases of the circulatory system	11	8	11	8	10	7	8	7	8	9	8	8	100 (489)	0.095
J00–J99	Diseases of the respiratory system	4	7	7	6	6	8	7	10	7	13	10	16	100 (304)	0.007
K00–K93	Diseases of the digestive system	8	7	10	8	9	8	7	8	4	12	9	9	100 (295)	0.678
L00–L99	Diseases of the skin and subcutaneous tissue	5	5	11	5	0	5	11	11	5	16	11	16	100 (19)	0.036
M00–M99	Diseases of the musculoskeletal system and connective tissue	8	3	5	14	14	5	16	11	3	5	8	8	100 (37)	0.940
N00–N99	Diseases of the genitourinary system	5	8	0	5	5	7	7	8	8	17	18	11	100 (83)	0.014
O00–O99	Pregnancy, childbirth, and the puerperium	0	0	29	0	0	14	14	0	14	0	14	14	100 (7)	0.520
Q00–Q99	Congenital malformations, deformations, and chromosomal abnormalities	0	0	0	0	0	0	0	100	0	0	0	0	100 (1)	0.664
R00–R99	Symptoms, signs, and abnormal clinical and laboratory findings, not elsewhere classified	7	6	8	8	7	7	7	6	10	11	10	13	100 (457)	0.013
S00–T98	Injury, poisoning, and certain other consequences of external causes	23	28	23	26	19	22	17	26	27	22	26	20	100 (756)	0.304
Medical	Medical (T36-T69, T80-T98)	4	8	7	7	5	5	4	8	5	7	7	7	100 (201)	0.098
Trauma	Trauma (S00-T35, T70-T79, T90-T99)	19	20	16	19	14	17	13	18	22	15	19	13	100 (555)	0.270
Z00–Z99	Factors influencing health status and contact with health services	7	13	9	11	9	6	8	8	8	5	5	4	100 (245)	0.104
Total % (n)		7 (242)	8 (246)	9 (281)	8 (253)	8 (253)	7 (216)	7 (222)	8 (264)	8 (254)	11 (369)	10 (323)	11 (343)	100 (3,266)	

Cuzick’s test for trend^a^

### Mortality

**Fig. 6:**
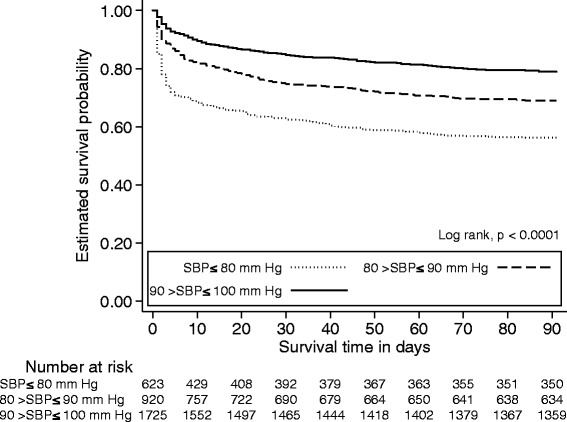
Kaplan-Meier curves illustrating overall 90-day survival according to different systolic blood pressure levels. Below the curves are listed the number at risk at corresponding intervals in survival time

The mean 7-day, 30-day and 90-day all-cause mortality was 15 % (95 % CI: 14–16), 22 % (95 % CI: 21–24) and 28 % (95 % CI: 27–30) respectively (Table 1). We observed an increase (*p* <0.001) in mortality among patients SBP ≤80 mm Hg (90-day mortality: 44 % (95 % CI: 40–48)) compared to patients with SBP between 90 >SBP ≤100 mm Hg (90-day mortality: 21 % (95 % CI: 19–23)). Kaplan-Meier curves are shown in Fig. 6 with the overall estimated probability of 90-day survival stratified into SBP intervals.

## Discussion

This study provides population-based epidemiological characteristics of adult hypotensive patients arriving to a University ED in Denmark. Our results showed that a first time presentation of hypotension was a common finding in the ED with an increasing annual trend in IRs throughout the period 2000–2011. By means of discharge diagnoses the etiology was clearly diversified and the 90-day all-cause mortality was 28 %.

Our primary aim was to address the IR and trend of hypotension in the ED. In our study we have reported an overall mean IR of SBP ≤100 mm Hg of 125/100,000 pyar. Comparing our IRs with other conditions suggest hypotension to be as common as first time hospitalization with myocardial infarction (MI) [12] and more common than ST-segment elevation MI [13]. While the IRs of MI have decreased during the past decades [12, 13] registered sepsis is on the rise [3]. The IRs of hypotension, in our study, increased in the years 2009–2011 compared to the years before. The interpretation of this increase is not obvious and may be confounded by factors such as greater awareness of critical illnesses and per se increased measurement of vital parameters.

We found higher IRs among elderly males, compared to women. Moreover 55 % of our cohort represented patients aged 65 years or more, increasing with decreasing SBP level. Accordingly, a large Canadian study analyzed 34,454 ED visits by older adults (>65 years), accounting for 22 % of the total ED visits in which, 74 % of patient visits were triaged as urgent or emergent [14]. The most common diagnoses (ICD-9 and ICD-10) were non-specific, relating to “symptoms, signs, and ill-defined conditions” (25 %). Injury and poisoning constituted 17 % of diagnoses, while diagnoses related to the circulatory system and respiratory system constituted 10 and 9 % of diagnoses, respectively [14].

Comparing these proportions with ICD-10 discharge groups in our study suggest a similar pattern, given the increasing IRs and the dominating ageing proportion of patients. Discharge diagnoses were dominated by injury and poisoning, circulatory system and unspecific diagnoses (symptoms and abnormal clinical/laboratory findings) in our cohort. Other studies, among undifferentiated non-traumatic hypotension in the ED, report sepsis and cardiovascular diseases as common etiologies [15, 16]. A similar etiological distribution applies for critically ill hypotensive patients in the ICU [1]. This difference could reflect the use of ICD-10 codes, and population-based setting, while others have applied primary clinical assessments and strict inclusion criteria when categorizing the etiology. Interestingly, infectious and cardiovascular diseases increased by each decile decrease in SBP level, whereas trauma decreased accordingly. Moreover, we found an increase in discharge diagnoses of infectious and respiratory diseases, disease of the genitourinary system, as well as symptoms, signs, and abnormal clinical and laboratory findings, not elsewhere classified. As a supplementary analysis we applied validated discharge diagnoses for patients with community-acquired infections presenting to the ED (see Appendix: Table 4 for ICD-10 codes validated by Henriksen et al. [17]). Using this algorithm we found a proportion of 13 % with a discharge diagnoses of infection, compared to 5 % in the initial analysis. The difference reflects our use of merely major ICD-10 groups, as certain infectious diseases (e.g. ICD10-J189 = “*pneumonia, unspecified”*) are grouped under respiratory diseases in the ICD10 system. Applying the validated discharge diagnoses for infections we found a confirmatory increasing trend (*p* = 0.011). Although the data source used is considered a unique information source to carry out epidemiological studies and health service research in our country, the discharge diagnoses among hypotensive ED patients have not undergone validation. The heterogeneous etiological data presented here, should therefore be interpreted bearing this in mind.

An important finding in this study is the 90-day all-cause mortality of 28 %. Correspondingly, in-hospital mortality among non-traumatic hypotensive patients, (SBP ≤100 mm Hg) is reported to be 10-25 % [16, 18–22] while mortality among traumatic hypotensive populations (SBP ≤100 mm Hg) are 7-24 % [23, 24]. As reported by Jones et al. we find an exposure of a single episode of hypotension (<100 mm Hg) in the ED setting to portend a possible later adverse outcome [7]. Furthermore, the mortality seem to increase with each decile decrease in SBP as reported previously [19].

We decided to include patient with a first time presentation of SBP ≤100 mm Hg measured within 3 h upon arrival. Only 85 patients did not meet this eligibility criterion. Moreover 92 % had their vital values measured within 30 min. All patients in our cohort had a mean SI ≥0.9 suggesting possible acute or critical illness. This could imply, that a great proportion of patients presented with clinical symptoms suggesting critical illness and therefore the ED personal deemed SBP measurement appropriate in order to delineate the hemodynamic stability. Whether a large proportion of our cohort presented with shock (e.g. organ failure or elevated lactate) is of interest, but not feasible based on the available data presented here.

We believe this population-based study provides robust data on the incidence of hypotension in the ED. When hypotension is present, mortality is substantial. Correct diagnosis and resuscitation of patients with hypotension are well-known steps to improve prognosis. Future epidemiological perspectives for research should address the underlying etiology and prognosis of undifferentiated hypotension as this could delineate targeted interventions at ED arrival. At the level of triage, SBP ≤100 mm Hg should be regarded as a critical finding and the cause of hypotension explored. Future prehospital protocolised management by combining e.g. ultrasound, vital parameters and lactate could further expedite resource allocation and triage of these, often critically ill patients.

### Study strengths and limitations

The Danish public healthcare system, with a complete, independently and prospectively recorded medical history, reduced the possible risk of information biases and loss to follow up was not an issue. With the use of the Danish population-based registries we were able to compute quite accurate estimates on the outcomes: incidence, mortality and comorbidity. We chose to use the first contact with hypotension in order minimize bias from repeated measurements. Furthermore we excluded patient with residency outside the catchment area and a previously reported admission with SBP ≤100 mm Hg in the years 1998–99 in order to avoid possible overestimation of the IRs.

Several issues and limitations should be considered when interpreting our results. Our single center design limits the generalizability of our findings. Although our ED is the only on serving this part of Denmark, the presence of “market share” within the bordering of other ED catchment areas in Denmark is a possibility. We are not able to adjust for possible hypotensive patients living in our catchment area, who have had contact to other hospitals. However, we have excluded patients living in municipalities outside of our ED catchment area and thereby minimized this proportion (*n* = 516, Fig. 1). The blood pressure measurements were registered prospectively and as a routine documentation and not necessarily for research purposes. However, a great proportion of cases did not have a SBP measured and registered at arrival (*n* = 273,794). These patients suffered from minor complaints where the nurse did not judge a SBP measurement relevant. Patients with medical complaints and trauma severe enough to warrant a SBP measurement are therefore the population of relevance. This must be kept in mind when interpreting our findings.

We acknowledge the possible limitation in the blood pressure measurement as the accuracy of the automatic oscillometric devices and measurement by auscultation can be inaccurate [25]. However, this is still the method used in most clinical and research based settings when describing blood pressure and we therefore find it generalizable.

We further acknowledge the limitations of the etiological characteristics. Ideally, a classification into shock categories could be clinical useful. However, these data were not available in the current dataset. We had missing values on covariates; ICD-codes (2 cases) and HR (128 cases), but not on SBP. Of notion, is the drop in the IR of 2008, which was caused by an organizational change in the electronic registration of vital parameters in this year.

Finally, our study and results can be influenced and confounded by unmeasured variables such as use of cardio-therapeutic medications known to inhibit the cardiovascular compensatory response in individuals and potentially mask hypotension and bias our estimates, especially among elderly comorbid patients using these medications. During the observation period a physician-staffed mobile emergency care unit was deployed (October 2007) in the pre-hospital setting. Accordingly, increased awareness and change in treatment algorithms in certain critical conditions have been introduced (surviving sepsis campaign and percutaneous coronary intervention of myocardial infarction). Although we consider this proportion minimal, the possibility of patients suffering time-dependent illnesses diagnosed prehospitally (e.g. ruptured aneurism, myocardial infarction) and referred directly to a facility within our hospital (e.g. operational theatre or ICU) and thereby bypassing our ED is a possibility we acknowledge. Although there was no structural change in the primary care service, a change in general practitioners’ interest in assessing acute clinical conditions (due to the increasing specialization and fragmentation of primary care services) is another possibility we acknowledge.

## Conclusion

We conclude that a presentation with hypotension is a common critical finding among ED patients with an increasing trend. Adverse outcome are substantial carrying a 90-day all-cause mortality of 28 %. Using ICD-10 codes, etiological characteristics are diversified both at ED arrival and at hospital discharge. Prospective risk stratified protocols should evaluate the use and impact prognostics of hypotension in triage algorithms, both prehospitally and in the ED setting.

## Appendix

**Table 4:** ICD-10 codes identifying infections

Site of infection	ICD-10 discharge diagnosis
Central nervous system	A39.0, A39.2A, A86.9, A87.0, A87.9, B00.3, B00.4, B02.0, B02.2, B02.2A, B02.2B, B91.9, G00.1, G00.8, G00.9, G00.9A, G01.9, G04.0, G04.2, G06.0, G06.0 F, G06.2, G07.9
Lower respiratory tract	Pneumonia: A31.0A, A48.1, B37.1, J12.0, J13.9, J14.9, J15,J15.0, J15.1, J15.2, J15.4, J15.5, J15.7, J15.8, J15.9, J17.0, J17.8C, J18, J18.0, J18.1, J18.8, J18.9, J20.9, J20.9A, J21.9, J22.9, J69.0, J69.8, J69.8A Other: A15.0, A15.1, A15.2, A15.9, B90.9, J40.9, J44.0, J85.1, J85.2, J86.0, J86.9
Urinary tract	A41.9B, N10.9, N12.9, N13.6, N30.0, N30.8, N30.9, N39.0, N39.0B
Abdominal	A00.9, A01.1, A02.0, A03.8, A04.3, A04.5, A04.7, A04.8, A04.9, A05.9, A08.0, A08.1, A08.2, A08.3, A08.4, A08.5, A09, A09.9, B67.0, K35.0, K35.0A, K35.1, K35.1A, K35.9, K57.2B, K57.3, K57.3A, K57.3B, K57.3 F, K57.9A, K65.0,K65.0A, K65.0G, K65.0 J, K65.8, K65.8I, K65.9, K75.0, K80.3, K80.4, K81.0, K81.9, K83.0
Cardiovascular	I30.0, I30.1, I30.8, I30.9, I33.0, I33.9, I38.9, I39.8
Skin, muscles, bones	A46.9, B00.1A,B00.1B, B37.2, K61.0, K61.0A, K61.1, K61.2, L02.2, L02.2 T, L02.4, L02.4 F, L02.4 K, L02.9, L02.9A, L03.1, L03.1E, L03.3, L08.8, L08.9, M00.0, M00.2, M00.2A, M00.8, M00.9, M46.3, M46.4, M46.5, M46.5A, M46.9, M71.1, M86.1, M86.8, M86.9
Viral/systemic	A51.5, A79.9, B00.1, B05.9, B20.4, B20.6, B20.8, B23.0, B23.2, B24.9, B25.8, B25.9, B27.0, B27.9, B50.9, B52.9, B54.9, B55.0, B58.9, J09.1, J09.9, J10.0, J10.8, J11, J11.0, J11.1, J11.8
Unknown	A40.1, A40.3, A40.8, A40.9, A41.0, A41.1, A41.1A, A41.2, A41.3, A41.4, A41.5, A41.8, A41.9, A49.9A, B37.7, A32.9, A41.9A, A42.9, A44.9, A48.2, A49.0, A49.1, A49.3, A49.8, A49.9, A68.9, A70.9, A81.2, B00.8, B02.9, B34.0, B34.9, B36.9, B37.0, B37.8, B80.9, B89.9, B95.5, B95.6, B95.6A, B96.4, B96.5, B96.8, B99.9, R50, R50.0, R50.8, R50.9, T81.4D, T84.6, T89.9
Other sites of infection	B00.2A, B02.3G, B37.3A, B37.4, B37.8C, E06.0, E06.1, H65.1, H66.0, H66.9, J00.9B, J01.0, J01.1, J01.2, J01.8, J01.9, J02.0, J02.9, J02.9B, J03.0, J03.9, J03.9A, J04.0, J05.1, J06.9, J36.9, J39.0C, K04.0A, K05.3A, K10.2C, K11.2C, K12.1, K62.8 L, N41.2, N45.0B, N45.9, N45.9A, N76.4A, O86.8

## Abbreviations

CCI: Charlson comorbidity index
CI: Confidence interval
ECG: Electrocardiography
ED: Emergency department
ICD-10: International classification of diseases, tenth revision
IR: Incidence rates
IQR: Interquartile range
MI: Myocardial infarction
HR: Heart rate
PRN-number: Personal registry number
Pyar: Person-years at risk
SBP: Systolic blood pressure
SI: Shock index
